# Functional magnetic resonance imaging (fMRI) item analysis of empathy and theory of mind

**DOI:** 10.1002/hbm.24966

**Published:** 2020-03-02

**Authors:** Matthias G. Tholen, Fynn‐Mathis Trautwein, Anne Böckler, Tania Singer, Philipp Kanske

**Affiliations:** ^1^ Centre for Cognitive Neuroscience, Department of Psychology University of Salzburg Austria; ^2^ Edmont J. Safra Brain Research Center University of Haifa Israel; ^3^ Department of Psychology Leibniz University Hannover Hannover Germany; ^4^ Max Planck Society, Social Neuroscience Lab Berlin Germany; ^5^ Clinical Psychology and Behavioral Neuroscience Faculty of Psychology, Technische Universität Dresden Dresden Germany; ^6^ Max Planck Institute for Human Cognitive and Brain Sciences Research Group Social Stress and Family Health Leipzig Germany

**Keywords:** affect sharing, anterior insula, mentalizing, social cognition, temporoparietal junction

## Abstract

In contrast to conventional functional magnetic resonance imaging (fMRI) analysis across participants, item analysis allows generalizing the observed neural response patterns from a specific stimulus set to the entire population of stimuli. In the present study, we perform an item analysis on an fMRI paradigm (EmpaToM) that measures the neural correlates of empathy and Theory of Mind (ToM). The task includes a large stimulus set (240 emotional vs. neutral videos to probe empathic responding and 240 ToM or factual reasoning questions to probe ToM), which we tested in two large participant samples (*N* = 178, *N* = 130). Both, the empathy‐related network comprising anterior insula, anterior cingulate/dorsomedial prefrontal cortex, inferior frontal gyrus, and dorsal temporoparietal junction/supramarginal gyrus (TPJ) and the ToM related network including ventral TPJ, superior temporal gyrus, temporal poles, and anterior and posterior midline regions, were observed across participants and items. Regression analyses confirmed that these activations are predicted by the empathy or ToM condition of the stimuli, but not by low‐level features such as video length, number of words, syllables or syntactic complexity. The item analysis also allowed for the selection of the most effective items to create optimized stimulus sets that provide the most stable and reproducible results. Finally, reproducibility was shown in the replication of all analyses in the second participant sample. The data demonstrate (a) the generalizability of empathy and ToM related neural activity and (b) the reproducibility of the EmpaToM task and its applicability in intervention and clinical imaging studies.

## 1 INTRODUCTION

Aiming at elucidating the mechanisms underlying social understanding, human neuroscience research has extensively investigated the brain correlates of how we feel with (affective route) and know about others (cognitive route). The affective route allows for sharing others' emotions (empathy, affect sharing) (de Vignemont & Singer, 2006), for example, when vicariously sharing another person's sadness or grief. The cognitive route enables reasoning about others' mental states (Theory of Mind, ToM, mentalizing) (Frith & Frith, 2005; Premack & Woodruff, 1978), for example, when attributing another person's belief, desire or intention. Several meta‐analyses across different experimental approaches to both empathy and ToM have consistently described two distinct neural networks related to these functions. Core regions of the empathy related network are found in the anterior insula (AI), anterior cingulate/dorsomedial prefrontal cortex (ACC/DMPFC), inferior frontal gyrus (IFG) and dorsal portions of the temporoparietal junction/supramarginal gyrus (TPJ/SMG) (Bzdok et al., 2012; Lamm, Decety, & Singer, 2011). The ToM related network includes the ventral TPJ, anterior and posterior medial prefrontal cortex (MPFC), superior temporal gyrus/sulcus (STG/STS), and temporal poles (Bzdok et al., 2012; Schurz, Radua, Aichhorn, Richlan, & Perner, 2014). Direct contrasts of both functions confirmed these networks with functional (Kanske, Böckler, Trautwein, & Singer, 2015) and structural neuroimaging (Eres, Decety, Louis, & Molenberghs, 2015; Valk et al., 2017; Valk, Bernhardt, Bockler, Kanske, & Singer, 2016). These studies show that empathizing and mentalizing engage distinct neural networks. Furthermore, brain regions also differ in cortical thickness according to the subjects' capacity to share emotions or to reason about mental states. Importantly, even though both functions are essential elements of higher‐level social processing, they are not directly related. The independence of empathy and ToM processing was demonstrated on the behavioral and the neural level (Kanske, Böckler, Trautwein, Parianen Lesemann, & Singer, 2016).

With three notable exceptions (Bruneau, Dufour, & Saxe, 2013; Dodell‐Feder, Koster‐Hale, Bedny, & Saxe, 2011; Theriault, Waytz, Heiphetz, & Young, 2017), all previous empathy and ToM investigations used conventional functional magnetic resonance imaging (fMRI) analyses across participants. These analyses allow generalizing the observed neural response patterns from the investigated participant sample to the human population they were sampled from, if they treat subjects as random‐effect (as has become standard since the late 1990s (Friston, Holmes, & Worsley, 1999)). However, the “fixed‐effect fallacy” still applies to the item‐level (Clark, 1973), that is, it is unsubstantiated to claim that activation patterns observed for a sample of stimuli would generalize to the population of stimuli, for instance, that the activity observed in an experiment eliciting emotional responses would generalize to the population of emotion‐eliciting stimuli. Furthermore, treating items as fixed could give single items with extreme responses disproportionate weight, thereby rendering a contrast of two conditions significant, just because a (possibly small) subset of items in one condition shows very strong activity, while the majority of items shows no effect. To overcome these problems, item analyses that treat items as random are common in many behavioral fields of study and have been shown to be feasible for fMRI analyses as well (Andrews‐Hanna, Reidler, Sepulcre, Poulin, & Buckner, 2010; Bedny, Aguirre, & Thompson‐Schill, 2007; Dodell‐Feder et al., 2011; Theriault et al., 2017; Troiani, Stigliani, Smith, & Epstein, 2014; Yee, Drucker, & Thompson‐Schill, 2010). Thus, Theriault et al. (2017) demonstrated positive correlations between regions in the ToM network and subjectivity ratings of metaethical judgments. Dodell‐Feder et al. (2011) replicated a subject‐wise analysis with an item analysis showing generalizability for false‐belief ToM stories. Bruneau et al. (2013) performed an item‐analysis on brief stories of physical or emotional pain yielding activity in the typical empathy and medial parts of the ToM related neural networks, respectively. This study did not, however, compare these results with the subject‐wise analysis published previously, which would directly show replicability of subject‐ and item‐wise analyses (Bruneau, Pluta, & Saxe, 2012). Interestingly, Bedny et al. (2007), who studied word class processing, found different results for subject‐ and item‐wise analyses, demonstrating the potential of item analysis to make theoretically important distinctions, which in that case reconciled conflicting evidence regarding the role of the prefrontal cortex in processing nouns vs. verbs (Bedny & Thompson‐Schill, 2006; Davis, Meunier, & Marslen‐Wilson, 2004; Shapiro, Moo, & Caramazza, 2006; Tyler, Bright, Fletcher, & Stamatakis, 2004).

In the present study, we aimed to investigate whether item‐analyses of empathy and ToM replicate the neural networks observed with subject‐wise analyses. To this end, we applied a previously validated fMRI paradigm that assesses both functions (EmpaToM) (Kanske et al., 2015). Empathy is probed via video stimuli with brief autobiographical narrations that are highly emotionally negative or neutral. The negative emotional narrations included such diverse issues as traffic accidents, involuntary pregnancy, partnership problems, diverse somatic and mental diseases and disorders, betrayal and guilt, political violence, seeking refuge, rape, natural disaster, miscarriage, assault or burglary. These videos have been shown to elicit empathic responses on a subjective, peripheral physiological, and on a neural level. ToM reasoning is demanded in subsequent questions that either ask for the mental states of the narrator in the previous video or for factual reasoning about the events of the narration. The mental state questions included first and second order, true and false beliefs, preferences and desires, irony, sarcasm, metaphors, (white) lies, deception and faux pas. The empathy and ToM measures were validated in several behavioral and fMRI studies through correlations and activation overlap with established empathy (Socio‐affective Video Taks; Klimecki, Leiberg, Lamm, & Singer, 2013) and ToM tasks (False Belief Task; Dodell‐Feder et al., 2011, Imposing Memory Task; Kinderman, Dunbar, & Bentall, 1998) and additional overlap with meta‐analytical findings (Bzdok et al., 2012; Dodell‐Feder et al., 2011; Kinderman et al., 1998; Klimecki et al., 2013). Conceptually, it is important for social neuroscience to show that empathy related neural activity generalizes beyond patterns only attributable to very specific stimuli, and whether ToM tasks other than false‐belief tasks (Dodell‐Feder et al., 2011) also lead to generalizable brain activation. To illustrate this form of generalization, as in psycholinguistics, where an item‐analysis in an experiment on verb‐processing allows generalizing the results from the limited sample of verbs tested to the population of verbs in that language (e.g., Bedny et al., 2007), replicating the subject‐analysis results in the EmpaToM with an item‐analysis would allow generalizing to the population of empathy‐inducing and ToM‐demanding conversational situations. Given the breadth of the sampled situations in the EmpaToM (240 distinct videos and questions), testing generalizability may be challenging, but could also have particular impact.

Furthermore, a principal problem in subject‐analysis is that discrepancies between two experimental conditions beyond the intended difference are uncontrollable confounds. Item‐analysis, in contrast, allows specifically testing whether activations observed in a contrast of two conditions are actually due to unintended low‐level differences between the conditions (e.g., more or less movement when telling an emotionally negative compared to a neutral story) rather than the intended difference (e.g., negative vs. neutral emotion). As item‐specific activation patterns are obtained, they can be associated to the specific features of each item. Given that it is impossible to completely match emotional and neutral stimuli without erasing the difference in emotionality, ruling out the influence of such low‐level features is a crucial issue. With regard to ToM, because of the considerable overlap of ToM related activity with regions involved in language processing, particularly in the temporal cortex and TPJ (Friederici, 2011; Schurz et al., 2014), it is critical to rule out the possibility that linguistic differences account for the observed ToM effects. Dodell‐Feder et al. (2011) convincingly demonstrated this for false‐belief tasks, but it is important to test whether this holds for other language‐based ToM tasks as well.

Because the EmpaToM was designed to be used in extensive longitudinal designs, it includes five parallel sets of different videos and questions that allow the repeated testing of the same participants across time. To enable usage of the EmpaToM in clinical and other settings, where only small participant samples are available or participants can be scanned for a very limited amount of time only, an item analysis on this large stimulus set affords the chance to select the most effective items to create stimulus sets that provide the most stable and reproducible results.

Finally, a major criticism of fMRI studies has been the limited sample size that not only reduces the likelihood to detect true effects, but also reduces the chance that a statistically significant result reflects a true effect (Button et al., 2013). Therefore, the present study made use of a large sample of participants (*N* = 178) and checked for reproducibility of the results in a second sample (*N* = 130).

In sum, applying item‐analyses to an fMRI task probing empathy and ToM, the present study addresses several questions: (a) Will the item‐analyses replicate the neural networks underlying empathy and ToM as observed with subject‐wise analyses? This would argue for generalizability of the observed brain activation patterns to the respective stimulus classes (i.e., neutral and emotional autobiographical video narrations; factual reasoning and ToM questions, the latter involving a variety of ToM demands such as irony, higher order mental state inference, false beliefs, etc.). (b) Can activity in the observed neural networks be predicted by low‐level stimulus characteristics (i.e., number of sentences, words, syllables, characters, predicates, conjunctives, changes in tense, passive constructions, subclauses, and the amount of motion)? (c) Does the item‐analysis allow creating stimulus sets including the most effective items to provide the most stable and reproducible results? (d) Are all of the above described results replicable in the second independent participant sample?

## 2 METHODS

### 2.1 Participants

Two samples of 191 and 141 German‐speaking participants were tested in the context of a large‐scale longitudinal study at baseline (ReSource Project; (Singer et al., 2016)).^1^ Participants were recruited from the general public through adverts. Recruitment of Sample 1 took place in 2012–2013 and of Sample 2 in 2013–2014. Participants had a very good language proficiency and were not included if they were below 20 or above 55 years of age, fulfilled the criteria for a mental or neurological disorder (according to structured clinical interviews for DSM‐IV axis I and axis II disorders; Wittchen, Zaudig, & Fydrich, 1997) or had any contraindication for MRI scanning. Twenty‐four participants had to be excluded due to study dropout (*N* = 5), dropout from MRI measurements (*N* = 1), or missing data due to technical, scheduling, or health issues (*N* = 18).

For Sample 1, 13 participants were excluded yielding a final sample of 178 participants (age mean = 40.9 years, *SD* = 9.5, 106 female). For Sample 2, 11 participants were excluded yielding a final sample of 130 participants (age mean = 40.4 years, *SD* = 9.0, 72 female).

The study was approved by the Research Ethics Committee of the University of Leipzig, number 376/12‐ff and the Research Ethics Committee of the Humboldt University in Berlin, numbers 2013‐02, 2013‐29, and 2014‐10. The study was registered with the Protocol Registration System of ClinicalTrials.gov under the title “Plasticity of the Compassionate Brain” with the ClinicalTrials.gov Identifier: NCT01833104. All participants signed informed consent prior to participation.

### 2.2 Stimuli and task

**Figure 1:**
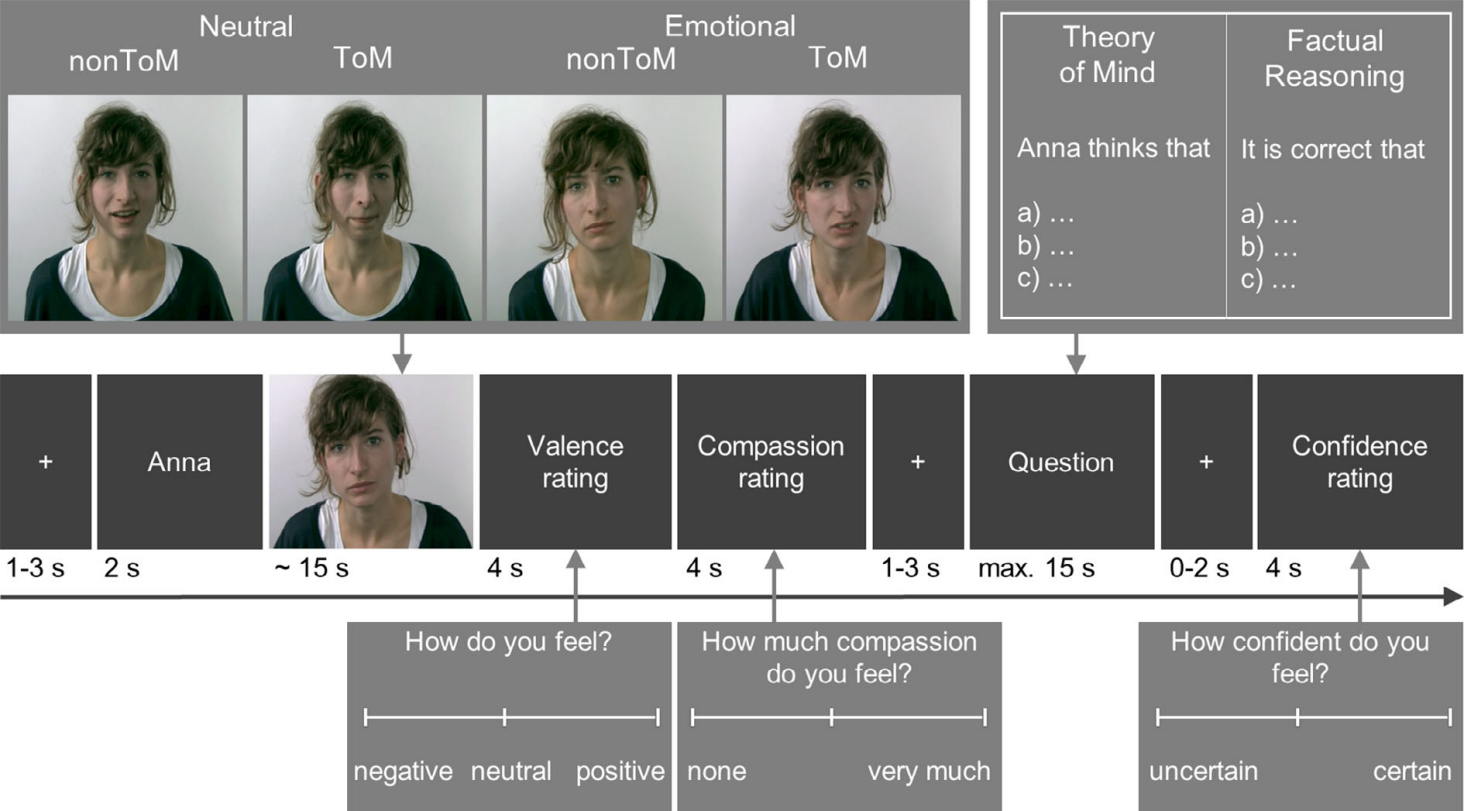
EmpaToM trial sequence. Emotional and neutral videos with and without ToM demands (2 × 2 design) are followed by valence and compassion ratings, ToM and factual reasoning questions, and a confidence rating (adopted from Kanske et al. (2015)). This study investigated the effects of subject‐ and itemwise analyses on the empathy and theory of mind contrasts. Empathy was tested via emotionally negative versus neutral videos and theory of mind was tested via mental state versus factual reasoning questions. ToM, Theory of Mind

For details of the EmpaToM task see (Kanske et al., 2015) (Figure 1). Each trial started with a fixation cross (1–3 s), followed by the name of a person (2 s), who would speak in the subsequent video (~15 s). Each participant was presented with videos of 12 persons, telling four different stories each that corresponded to four conditions (2 × 2 factorial design, negative vs. neutral emotion, ToM vs. no ToM demands). After this, participants rated the valence of their current emotional state (sliding scale from negative to neutral to positive; 4 s) and how much compassion^2^ they felt for the person in the previous video (sliding scale from none to very much; 4 s). A second fixation cross (1–3 s) was followed by a multiple choice question with three response options (one correct). These questions demanded either the attribution of mental states or factual reasoning (ToM vs. factual reasoning). Participants had to respond within 14 s. For example, stories and questions, see Data S1. After a third fixation cross (0–2 s), participants were asked to rate their confidence, that their decision was done correct (4 s) to allow assessing metacognitive abilities (Molenberghs, Trautwein, Bockler, Singer, & Kanske, 2016; Valk et al., 2016). In the present study we focused on the main empathy and ToM measures, that is, comparing emotional with neutral videos and ToM with factual reasoning questions (see (Kanske et al., 2015) for a validation of these contrasts).

**Figure 2:**
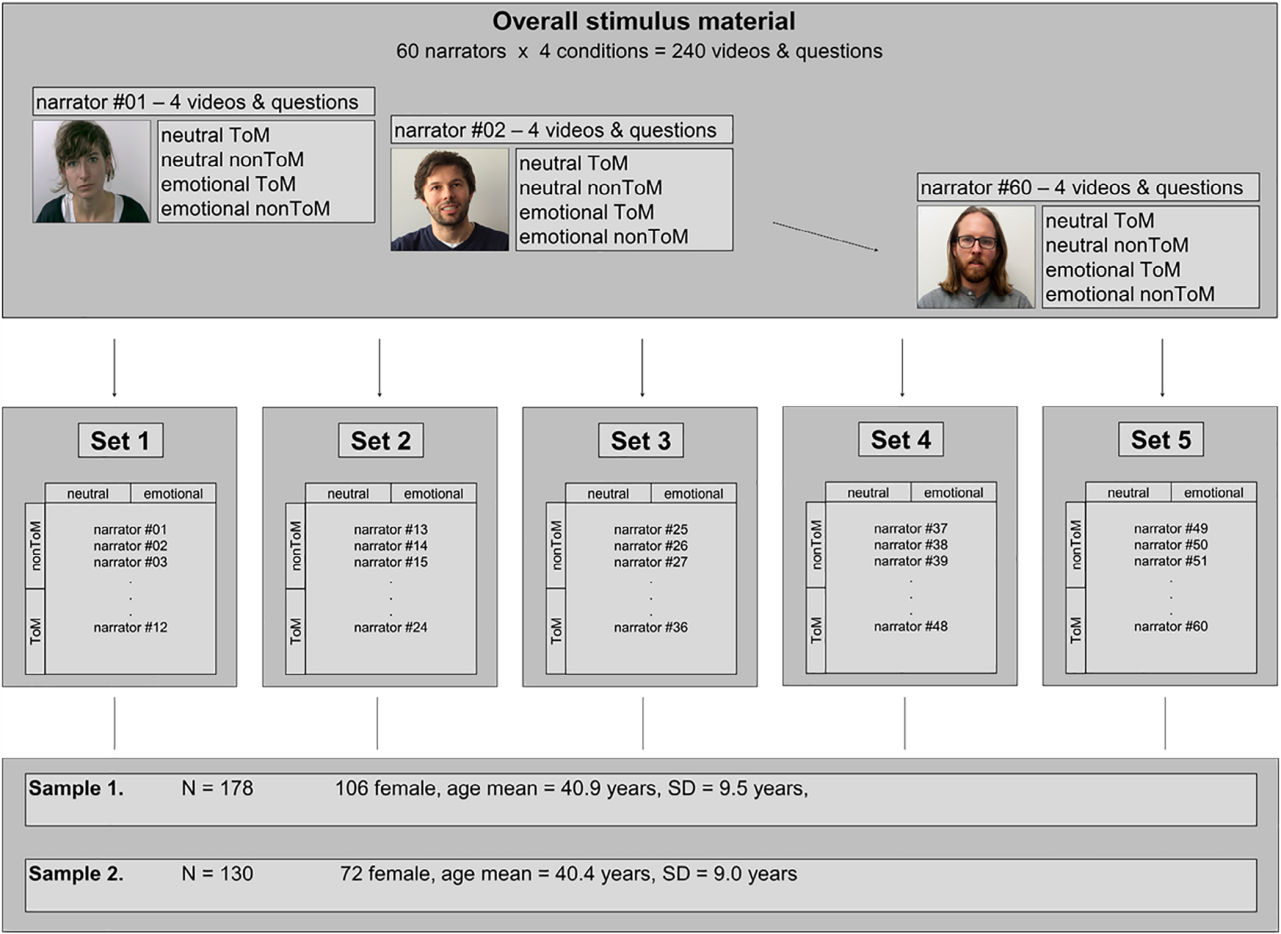
EmpaToM stimulus material. The overall stimulus material of the EmpaToM task contains 240 videos and questions with 60 different narrators in 4 conditions (emotional vs. neutral, ToM vs. nonToM), allocated to one of five parallel subsets. Each subset contains 12 different narrators in 4 conditions. The subsets are matched with regard to affect ratings, concern ratings, RTs, errors, confidence ratings, video lengths, and linguistic characteristics of the questions (number of words, characters, predicates, changes in tense, complexity of the sentences, number of passive sentence constructions, and number of conjunctives) (see Kanske et al., 2015). Subjects were randomly assigned to one of the five subsets, so that each subset was seen by a fifth of the participants in Sample 1 (*N* = 178) and Sample 2 (*N* = 130). ToM, Theory of Mind

The total stimulus set of the EmpaToM task comprised 240 videos and questions showing 60 different narrators in 4 conditions (see Figure 2). Based on this set, five parallel versions were created that each contained a different set of 12 narrators in 4 conditions (yielding 48 different videos and questions per set). The parallel sets were matched with regard to affect ratings, concern ratings, RTs, errors, confidence ratings, video lengths and linguistic characteristics of the questions (number of words, characters, predicates, changes in tense, complexity of the sentences [number of main and subordinate clauses], number of passive sentence constructions, and number of conjunctives), see (Kanske et al., 2015)). The five sets were randomly assigned to the participants such that each set (of 48 videos and questions) was seen by a fifth of the participants in Samples 1 and 2.

### 2.3 MRI data acquisition

Data were acquired on a 3 T MRI scanner (Siemens Magnetom Verio, Siemens Medical Solutions, Erlangen, Germany) using a 32 channel head coil. Functional images were acquired with a T2*‐weighted echo‐planar imaging (EPI) sequence (TR = 2,000 ms; TE = 27 ms, Flip Angle 90°, matrix = 70 × 70 mm, FOV = 210 mm). Within one TR, 37 axial slices of 3 mm were acquired. In addition, we collected a high‐resolution structural image (1 × 1 × 1 mm) with a T1‐weighted MPRAGE sequence.

### 2.4 Behavioral data analysis

Repeated measures analyses of variance were calculated across subjects and across items. In particular, we contrasted valence ratings after emotional and neutral videos as an indicator of empathic responding and analyzed performance (RTs and accuracies) after ToM questions as an indicator of ToM capacity. Each subject contributed ratings and performance measures in these conditions, averaged across all items. Complementarily, each item (i.e., narrator, each of which told four different stories) contributed measures in each condition, averaged across all participants.

### 2.5 fMRI data analysis

Data preprocessing and statistical analyses were performed with SPM 8 (http://www.fil.ion.ucl.ac.uk/spm) running in a MATLAB 7.6 environment (Mathworks Inc., Sherbon MA). Functional images were coregistered to the SPM single‐subject canonical EPI image, slice‐time corrected and realigned to the mean image volume for motion correction. The high‐resolution structural image was coregistered to the SPM single‐subject canonical T1 image and then to the average functional image. Normalization parameters of the structural image into the Montreal Neurological Institute (MNI) space were used for spatial normalization of the functional images. These images were resampled to isotropic 3 × 3 × 3 mm voxels and smoothed with an 8 mm FWHM Gaussian Kernel.

The statistical analyses were performed by using the general linear model. For the subject‐wise analysis, onset and duration of the four video types and their corresponding questions were modeled. These regressors were convolved by a canonical hemodynamic response function. Six regressors accounting head movement effects were modeled as covariates of no interest. RobustWLS Toolbox (Diedrichsen & Shadmehr, 2005) was used to reduce potential noise‐artifact. Contrast images for empathy (emotional vs. neutral videos) and ToM (ToM vs. nonToM questions) were calculated by applying linear weights to the parameter estimates and entered into one‐sample *t*‐tests for random effects analysis.

The item analyses were performed for each contrast separately by modeling the emotional and neutral videos, and the ToM and factual reasoning questions on the individual subject level. Each analysis resulted in 48 beta maps per subject (12 narrators × 4 conditions). The beta maps were averaged across the subjects within the five parallel versions (see Figure 2) to receive one single beta map per narrator and condition. For each of the five subgroups, this method yielded 48 beta maps at which each beta map comprised a mean beta value across subjects at every voxel, adding up to 240 beta maps in total. For the second‐level random effects analyses, we modeled the main contrasts between the condition differences (emotional vs. neutral videos, ToM vs. nonToM questions) together with the factor of subgroups as covariates of no interest in order to account for the dependencies between the 240 beta maps corresponding to the five groups of participants. The main contrasts were tested with two sample *t*‐tests.

The results for the subject‐wise as well as the item‐wise analyses were thresholded at *p* < .001 at voxel‐level together with an FWE (family‐wise error) correction (*p* < .05) at the cluster level.

### 2.6 Regression analysis

For both contrasts, regions of interest (ROI) (*N* = 46, 23 ToM, 23 empathy) were defined on the basis of the subject‐wise random effects analyses of Sample 1 (see Table 1 for empathy, Table 2 for ToM). They were used to extract the beta values from Sample 2 for the respective contrasts and consisted each of a sphere of contiguous voxels, 5 mm in radius. This procedure has two advantages: First, by using the ROIs from the subject‐wise analysis, we might be able to explain differences between item‐ and subject‐wise analyses that are due to low‐level features. Second, the data of the regression analysis is based on independently defined ROIs. To test whether the activations can be additionally explained by linguistic factors each item was coded by at least two researchers in 9 different features. They comprised the following set of variables and were coded for each of the stories (spoken text, empathy contrast) and questions (written text, ToM contrast): number of words, characters, sentences, syllables (as measures of the amount of spoken or written text), predicates, tenses, passives, conjunctives and complexity (as measures of syntactic difficulty). Additionally, for the empathy contrast three general features were coded to characterize the video material: duration of the video, motion and velocity of the narrator's movement. In the following three passages, we further illustrate the choice of these features.

**Table 1:** Whole brain subject‐ and item‐wise random effects results for Videos Emotional > Neutral. The results are reported at a voxel‐level threshold of *p* < .001 uncorrected together with an FWE‐corrected cluster threshold of *p* < .05

	*H*	MNI coordinates					
		*x*	*y*	*z*	*T*	*Z*	Cluster
*Subject‐wise Group#1*							
Inferior frontal gyrus	*L*	−48	39	−9	10.68	>8.21	1,027
Middle frontal	*L*	−42	15	45	10.04	>8.21	
Anterior insula	*L*	−36	21	−6	8.58	7.82	
Superior medial frontal cortex	*L*	−3	33	51	10.53	>8.21	1,257
Superior medial frontal	*R*	9	21	57	8.69	>8.21	
Inferior frontal gyrus	*R*	51	30	−6	10.01	>8.21	737
Middle frontal	*R*	42	21	39	6.96	6.53	
Anterior insula	*R*	30	24	−15	6.64	6.27	
Ventral striatum	*R*	9	3	0	6.29	5.97	153
Ventral striatum	*L*	−6	−3	0	6.16	5.86	
Caudate	*L*	−12	6	12	6.12	5.82	
Caudate	*R*	12	6	12	6	5.82	
Middle cingulate		0	−18	39	8.25	7.58	82
Middle temporal cortex	*L*	−54	−30	−12	6.08	5.79	26
TPJ‐angular/supramarginal gyrus	*R*	63	−48	33	9.71	>8.21	448
Middle temporal cortex	*R*	60	−57	9	7.44	6.93	
TPJ‐angular/supramarginal gyrus	*L*	−54	−51	33	12.49	>8.21	599
Precuneus		0	−63	36	12.01	>8.21	614
Lingual gyrus	*L*	−6	−75	−3	8.07	7.43	162
Middle occipital	*R*	42	−84	18	5.98	5.7	30
Middle occipital	*L*	−39	−90	9	5.1	4.92	13
Cerebellum	*L*	−15	−78	−30	9.88	>8.21	186
Cerebellum	*R*	18	−81	−33	10.04	>8.21	219
*Item‐wise Group#1*							
Anterior insula	*L*	−39	21	−9	9.83	>8.21	767
Inferior frontal gyrus	*L*	−45	42	−12	8.71	7.64	
Middle frontal	*L*	−42	18	42	10.64	>8.21	248
Superior medial frontal cortex		0	42	45	10.89	>8.21	1,239
Superior medial frontal	*R*	12	18	54	7.67	6.90	
Inferior frontal gyrus	*R*	45	27	−12	9.33	>8.21	492
Anterior insula	*R*	30	24	−12	8.19	7.27	
Middle frontal	*R*	39	24	39	6.68	6.14	148
Ventral striatum	*R*	9	3	0	6.33	5.86	59
Caudate	*R*	12	9	12	5.13	4.86	
Caudate	*L*	−12	12	15	6.64	6.11	54
Ventral striatum	*L*	−6	0	−3	5.70	5.35	
Middle cingulate		0	−18	39	7.00	6.39	45
Middle temporal cortex	*L*	−54	−30	−15	5.96	5.56	20
TPJ‐angular/supramarginal gyrus	*R*	60	−45	36	8.02	7.15	258
Middle temporal cortex	*R*	48	−48	18	5.12	4.86	
TPJ‐angular/supramarginal gyrus	*L*	−54	−51	30	10.64	>8.21	412
Precuneus	*L*	−6	−60	33	8.87	7.74	413
Lingual gyrus	*L*						
Middle occipital	*R*						
Middle occipital	*L*						
Cerebellum	*L*	−15	−78	−30	8.08	7.20	145
Cerebellum	*R*	15	−78	−30	8.35	7.39	199
*Subject‐wise Group#2*							
Inferior frontal gyrus	*L*	−45	36	−6	8.84	7.80	469
Anterior insula	*L*	−36	27	−3	7.27	6.64	
Middle frontal	*L*	−39	15	39	7.18	6.57	106
Middle frontal	*L*	−36	60	−3	5.76	5.42	19
Superior medial frontal cortex		0	45	33	10.64	>8.21	758
Superior medial frontal	*R*	15	21	63	5.18	4.93	
Inferior frontal gyrus	*R*	45	27	3	7.31	6.67	290
Anterior insula	*R*	30	21	−15	6.50	6.03	
Middle frontal	*R*	42	18	36	5.36	5.08	17
Ventral striatum	*R*	6	0	−3	6.00	5.62	32
Caudate	*R*	12	9	9	5.35	5.07	
Ventral striatum	*L*	−6	0	0	5.84	5.49	32
Caudate	*R*	−12	6	12	5.63	5.31	
Middle cingulate		0	−18	39	6.38	5.93	16
Middle temporal cortex	*L*						
TPJ‐angular/supramarginal gyrus	*R*	63	−51	24	7.93	7.15	153
Middle temporal cortex	*R*						
TPJ‐angular/supramarginal gyrus	*L*	−57	−51	33	10.36	>8.21	242
Precuneus	*L*	−6	−51	33	7.78	7.03	261
Lingual gyrus	*L*	−9	−75	−3	6.09	5.70	19
Middle occipital	*R*						
Middle occipital	*L*						
Cerebellum	*L*	−18	−78	−33	7.85	7.08	115
Cerebellum	*R*	24	−75	−33	7.90	7.12	96
*Itemwise Group#2*							
Inferior frontal gyrus	*L*	−45	42	−6	8.52	7.50	588
Anterior insula	*L*	−27	24	−9	7.81	7.00	
Middle frontal	*L*	−39	15	39	6.75	6.19	94
Superior medial frontal cortex	*L*	0	39	42	9.61	>8.21	823
Superior medial frontal	*L*	−3	33	48	9.34	>8.21	
Inferior frontal gyrus	*R*	42	33	−3	7.90	7.06	283
Anterior insula	*R*	33	21	−15	7.43	6.72	
Middle frontal	*R*	36	18	36	5.53	5.20	15
Caudate	*R*	6	−6	−12	6.91	6.32	55
Ventral striatum	*L*	9	0	−3	6.39	5.91	
Ventral striatum	*L*	−6	0	0	6.03	5.62	
Caudate	*R*						
Middle cingulate							
Middle temporal cortex	*L*						
TPJ‐angular/supramarginal gyrus	*R*	63	−51	27	5.66	5.31	40
Middle temporal cortex	*R*						
TPJ‐angular/supramarginal gyrus	*L*	−54	−51	33	9.92	>8.21	214
Precuneus	*L*	−6	−51	33	6.21	5.76	70
Lingual gyrus	*L*						
Middle occipital	*R*						
Middle occipital	*L*						
Cerebellum	*L*	−15	−78	−30	7.94	7.09	99
Cerebellum	*R*	15	−81	−30	7.42	6.71	108

Abbreviations: FWE, family‐wise error; TPJ, temporoparietal junction/supramarginal gyrus.

**Table 2:** Whole brain subject‐ and item‐wise random effects results for Questions ToM > non ToM The results are reported at a voxel‐level threshold of *p* < .001 uncorrected together with an FWE‐corrected cluster threshold of *p* < .05

	*H*	MNI coordinates					
		*x*	*y*	*z*	*T*	*Z*	Cluster
*Subject‐wise Group#1*							
Rectus	*R*	3	57	−18	7.71	7.15	38
Superior medial frontal	*L*	−9	54	24	13.72	>8.21	1,185
Superior frontal	*L*	−9	54	33	12.34	>8.21	
Superior medial frontal	*R*	9	57	21	11.73	>8.21	
Inferior frontal gyrus	*R*	54	30	3	6.24	5.92	52
Inferior frontal gyrus	*L*	−51	24	6	10.32	>8.21	226
Inferior frontal gyrus	*L*	−45	27	−9	9.93	>8.21	
Temporal pole	*R*	51	9	−33	14.68	>8.21	121
Temporal pole	*L*	−51	3	−30	12	>8.21	79
Postcentral	*L*	−54	−6	48	6.05	5.76	13
Middle cingulate		0	−15	39	8.56	7.81	50
Supplementary motor area	*R*	6	−24	57	5.37	5.17	10
TPJ‐middle temporal	*R*	51	−30	−3	10.61	>8.21	640
TPJ‐superior temporal	*R*	48	−18	−9	9.81	>8.21	
TPJ‐angular gyrus	*R*	63	−45	21	7.76	7.19	
Posterior cingulate/precuneus	*L*	−6	−51	30	16.38	>8.21	328
TPJ‐angular gyrus	*L*	−51	−57	24	15.81	>8.21	1,019
TPJ‐middle temporal	*L*	−48	−30	−3	10.49	>8.21	
TPJ‐superior temporal	*L*	−60	−18	−6	9.53	>8.21	
Cuneus	*L*	−9	−93	30	5.7	5.45	10
Cuneus	*R*	15	−87	39	6.11	5.82	24
Cerebellum	*L*	−27	−81	−36	14.65	>8.21	101
Cerebellum	*R*	27	−78	−33	15.82	>8.21	145
*Item‐wise Group#1*							
Superior medial frontal	*L*	−12	57	36	15.99	>8.21	1,241
Rectus	*R*	3	57	−18	7.97	7.12	
Superior frontal	*L*	−6	54	18	14.93	>8.21	
Superior medial frontal	*L*	−9	30	57	10.34	>8.21	
Inferior frontal gyrus	*R*	48	30	−9	6.39	5.91	32
Inferior frontal gyrus	*L*	−54	24	6	11.74	>8.21	243
Inferior frontal gyrus	*L*	−45	30	−9	10.62	>8.21	
Temporal pole	*R*	51	9	−33	16.30	>8.21	125
Temporal pole	*L*	−54	24	6	11.74	>8.21	243
Postcentral	*L*						
Middle cingulate		0	−12	39	8.63	7.58	39
Supplementary motor area	*R*						
TPJ‐middle temporal	*R*	48	−30	−3	10.40	>8.21	332
TPJ‐superior temporal	*R*	63	−51	21	6.42	5.93	71
TPJ‐angular gyrus	*R*	66	−42	24	5.77	5.40	
Posterior cingulate/precuneus	*L*	−9	−51	33	12.16	>8.21	253
TPJ‐angular gyrus	*L*	−51	−54	24	15.64	>8.21	889
TPJ‐superior temporal	*L*	−60	−15	−9	9.40	>8.21	
TPJ‐middle temporal	*L*	−48	−33	−6	8.82	7.71	
Cuneus	*L*						
Cuneus	*R*						
Cerebellum	*L*	51	9	−33	16.30	>8.21	125
Cerebellum	*R*	27	−78	−36	16.88	>8.21	135
*Subject‐wise Group#2*							
Rectus	*R*						
Superior medial frontal	*L*	−6	57	21	13.83	>8.21	1,008
Superior medial frontal	*R*	6	57	15	12.56	>8.21	
Supplementary motor area	*L*	−6	15	60	10.91	>8.21	
Inferior frontal gyrus	*R*	57	27	0	5.45	5.16	15
Inferior frontal gyrus	*L*	−48	27	0	10.69	>8.21	206
Temporal pole	*R*	51	12	−27	14.12	>8.21	147
Temporal pole	*L*	−51	9	−30	11.66	>8.21	446
Postcentral	*L*	−51	−6	51	5.93	5.56	15
Middle cingulate		0	−15	39	8.55	7.60	68
TPJ‐middle temporal	*R*	48	−27	−6	11.15	>8.21	446
TPJ‐angular gyrus	*R*	66	−45	18	6.52	6.05	
TPJ‐superior temporal	*R*	66	−36	24	6.42	5.97	
Posterior cingulate/precuneus	*L*	−6	−51	33	12.45	>8.21	251
TPJ‐angular gyrus	*L*	−45	−54	24	13.41	>8.21	778
TPJ‐middle temporal	*L*	−54	−27	−3	9.16	>8.21	
TPJ‐superior temporal	*L*	−63	−15	−15	6.43	5.98	
Cuneus	*L*	−9	−93	30	5.69	5.36	17
Cuneus	*R*						
Cerebellum	*L*	−27	−78	−36	13.97	>8.21	67
Cerebellum	*R*	30	−78	−36	14.07	>8.21	79
*Item‐wise Group#2*							
Rectus		0	51	−21	7.49	6.76	42
Superior medial frontal	*L*	−6	54	27	16.11	>8.21	1,213
Superior medial frontal	*R*	6	60	15	12.49	>8.21	
Superior medial frontal	*L*	−6	45	45	12.04	>8.21	
Inferior frontal gyrus	*R*	54	27	0	6.45	5.96	34
Inferior frontal gyrus	*L*	−45	30	−6	11.88	>8.21	264
Inferior frontal gyrus	*L*	−51	24	6	10.28	>8.21	
Temporal pole	*R*	51	12	−33	14.74	>8.21	164
Temporal pole	*L*	−48	12	−33	14.32	>8.21	97
Postcentral	*L*	−39	−21	21	5.68	5.33	14
Middle cingulate	*L*	−3	−12	39	7.18	6.52	45
Supplementary motor area	*R*						
TPJ‐middle temporal	*R*	45	−27	−6	11.24	>8.21	368
TPJ‐superior temporal	*R*	60	−54	24	6.79	6.23	
TPJ‐angular gyrus	*R*	66	−42	18	6.21	5.77	
Posterior cingulate/precuneus	*L*	−9	−51	33	12.14	>8.21	261
TPJ‐angular gyrus	*L*	−48	−57	27	13.33	>8.21	653
TPJ‐superior temporal	*L*	−48	−33	−3	9.77	>8.21	
TPJ‐middle temporal	*L*	−63	−18	−9	7.13	6.49	
Cuneus	*L*						
Cuneus	*R*						
Cerebellum	*L*	−24	−78	−36	12.79	>8.21	76
Cerebellum	*R*	27	−78	−36	13.29	>8.21	89

Abbreviations: FWE, family‐wise error; TPJ, temporoparietal junction/supramarginal gyrus.

The amount of spoken or written text, for example, measured by the number of words, has been used as a proxy for constituent size (Goucha & Friederici, 2015; Pallier, Devauchelle, & Dehaene, 2011). These studies showed that increasing constituent size is associated with an increase of neural activation in left hemispheric cortical areas such as the inferior frontal gyrus, temporo‐parietal junction, superior temporal sulcus and temporal pole, regions that are also engaged during empathy and theory of mind processing. Therefore, we tested whether differences in the number of words, characters, sentences or syllables can account for the observed effects in the EmpaTom task.

Five additional features measure aspects of syntactic complexity, that is, number of predicates, tenses, passives, conjunctives and complexity (lexical diversity: type token ratio). Syntactic complexity is correlated with working memory load indicated by higher error rates and longer processing times in sentence comprehension. FMRI studies showed that this effect modulates the neural activity in the left inferior frontal gyrus, middle frontal gyrus, and temporo‐parietal junction (Meltzer, McArdle, Schafer, & Braun, 2010; Newman, Malaia, Seo, & Cheng, 2013) suggesting the possibility that items with higher syntactic complexity influence activation patterns in the same cortical areas that are engaged during empathy and ToM processing.

Besides to low‐level features that are associated to spoken and written text, we additionally selected three general low‐level features that characterized the video material: duration of videos, motion and velocity of the narrator's movement. Emotionality may not only be communicated by language and facial expression but is also facilitated by spontaneous gestures and movements (Dick, Solodkin, & Small, 2010). Gesture comprehension is supported by a cortical network comprising the bilateral temporo‐parietal junction, bilateral superior parietal lobe, left inferior and middle frontal gyrus, and the left superior and middle temporal gyrus (Yang, Andric, & Mathew, 2015). Because of the considerable overlap with empathy related activity, we included these factors into the regression analysis to rule out that differences in the video material account for the observed empathy effects.

We performed stepwise forward/backward regression analyses with the item responses in the previously defined ROIs as dependent variables and condition and the selected features as independent variables. Stepwise regression is an iterative process of selecting and eliminating multiple variables depending on the model's best fit to the data. It is particularly useful in cases where there are large numbers of predictors. In each step, a predictor is added to the regression which most improves the fitting of the data (forward selection). To avoid overfitting, the predictors are excluded from the model if their contribution to predicting the outcome becomes non‐significant (backward elimination). We used rather strict entry and removal criteria that were based on the number of predictors to account for multiple testing (theory of mind (10 predictors): entry/removal: *p* = .005/*p* = .01; empathy (13 predictors): entry/removal: *p* = .0038/*p* = .0077). The analyses were performed on IBM SPSS Statistics for Windows, version 26.0 (IBM Corp., Armonk, NY).

### 2.7 Optimized sets of stimuli

The results of the item analyses were used to identify optimal sets of items which elicit the most prototypical response in both contrasts (empathy and ToM), that is, those items that produce the greatest activation in the theory of mind and empathy network. More specifically, we selected the items with the highest beta values in the experimental conditions and the lowest beta values in the control conditions for the regions of interest that were defined on the basis of the subject‐wise random effects analyses of Sample 1. We identified two sets, one with 48, the other with 40 videos and questions (see Data S2 and S3). Additionally, to allow for use in longitudinal designs, we identified two parallel sets of stimuli, that is, two sets with 48 and two sets with 40 videos and questions each (see Data S4 and S5). The sets with a reduced number of trials still reliably produce activations in the theory of mind and empathy network, and might therefore particularly be useful in clinical studies. The parallel sets are matched regarding on the extent to which they recruit the respective ROIs as well as to behavioral measures (affect, concern, confidence ratings and response time, accuracy in the questions), linguistic factors (number of words, characters, sentences, syllables, predicates, tenses, passives, conjunctives, and complexity) and general characteristics of the stimulus material (gender and age of narrator, movement and velocity, duration of the video).

## 3 RESULTS

### 3.1 Behavioral data

We first analyzed data from Sample 1. To test for emotion effects, we compared the valence ratings after emotional (*M* = −1.11, *SD* = .61) and neutral videos (*M* = .43, *SD* = .39), which yielded significant differences in subject‐ (*F*[1,177] = 794.29, *p* < .001) and item‐wise analyses (*F*[1,59] = 1,243.56, *p* < .001). To test, whether performance in ToM and nonToM questions differed, we compared RTs and accuracies in ToM (*M* = 8,450.58 ms, *SD* = 1,346.38; *M* = 64.05%, *SD* = 12.31, chance level = 33.33%) and nonToM questions (*M* = 8,490.93, *SD* = 1,272.54 ms; *M* = 55.05%, *SD* = 16.11, chance level = 33.33%). In RTs we found no significant differences in subject‐ (*F*[1,177] = .63, *p* > .40) and item‐wise analyses (*F*[1,59] < .001, *p* > .99). Accuracies, in contrast, were higher in the ToM than in the nonToM conditions in both subject‐ (*F*(1,177 = 69.20, *p* < .001) and item‐wise analyses (*F*[1,59] = 14.92, *p* < .001), indicating that the nonToM questions were slightly more difficult. Crucially, the subject‐ and item‐wise analyses were in line with each other for all measures.

The pattern of results was the same in Sample 2. For emotion effects, the valence ratings after emotional (*M* = −1.00, *SD* = .70) and neutral videos (*M* = .48, *SD* = .42), yielded significant differences in subject‐ (*F*[1,129] = 470.18, *p* < .001) and item‐wise analyses (*F*[1,59] = 937.13, *p* < .001). Performance in ToM (*M* = 8,471.71 ms, *SD* = 1,334.42; *M* = 67.14%, *SD* = 11.85, chance level = 33.33%) and nonToM questions (*M* = 8,563.97, *SD* = 1,329.51 ms; *M* = 57.43%, *SD* = 15.30, chance level = 33.33%) resembled Sample 1. RTs did not differ in subject‐ (*F*[1,129] = 2.40, *p* > .10) and item‐wise analyses (*F*[1,59] < .81, *p* > .35), but accuracies were higher in the ToM than in the nonToM conditions in both subject‐ (*F*(1,129 = 53.96, *p* < .001) and item‐wise analyses (*F*[1,59] = 16.08, *p* < .001). Again, the subject‐ and item‐wise analyses were perfectly in line with each other.

### 3.2 Neuroimaging data

#### 3.2.1 Empathy

**Figure 3:**
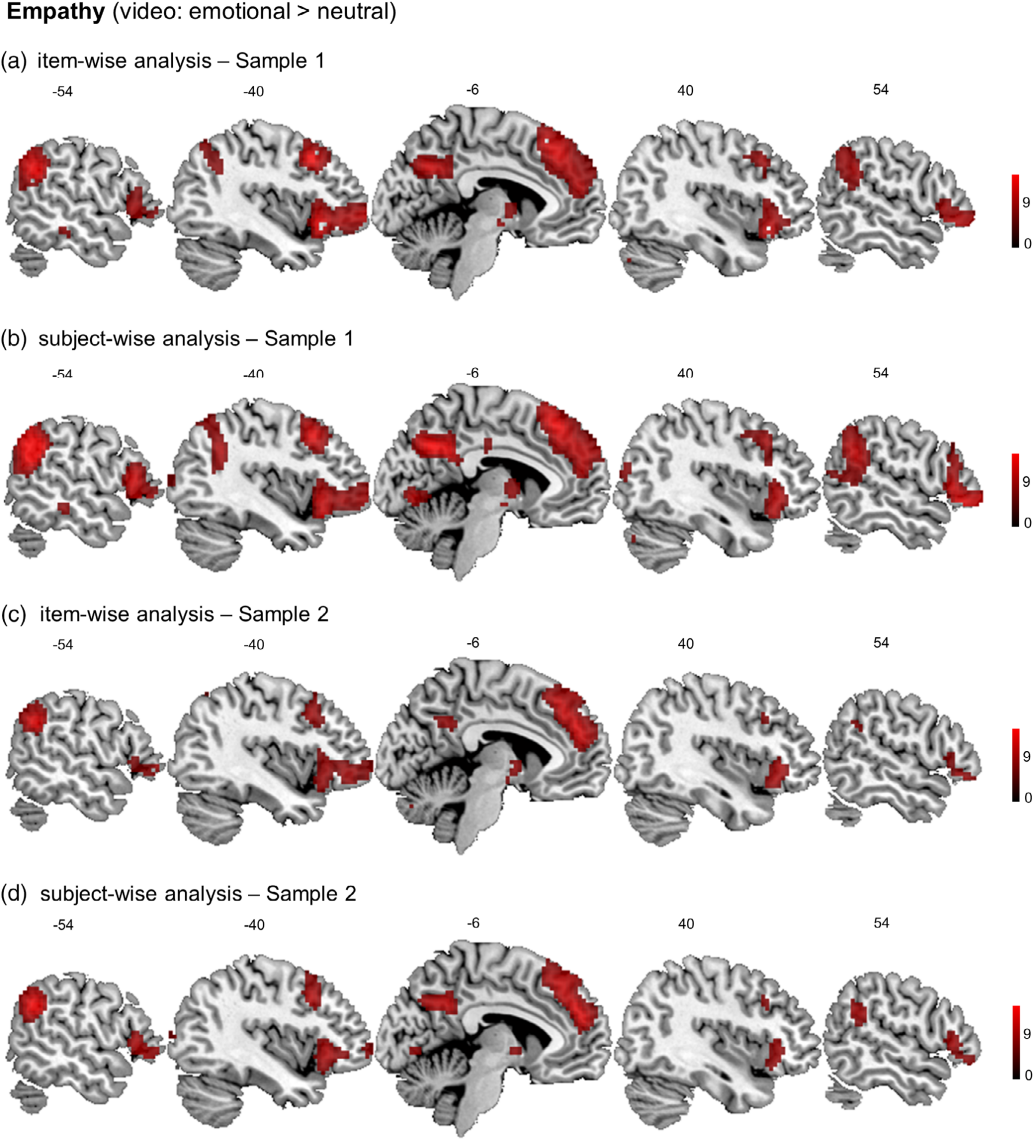
Consistency of the empathy related activation patterns (video: emotional > neutral) across item‐wise (a) and subject‐wise (b) analyses in Sample 1 (*N* = 178) and in Sample 2 (*N* = 130) (c, d, respectively). The results show activity in the empathy related network for emotional versus neutral videos, both across subjects and across items. This network includes anterior insula, anterior cingulate cortex/dorsomedial prefrontal cortex, inferior frontal gyrus and dorsal portions of the temporoparietal junction (supramarginal gyrus)

We performed whole brain subject‐ and item‐wise random effects analyses, first on the data set acquired in Sample 1 (see Figure 3a,b and Table 1). The results show activity in the typical empathy related neural network for emotional versus neutral videos, both across subjects and across items. This network includes bilateral AI, ACC/DMPFC, IFG, and dorsal portions of TPJ/SMG. A few regions showed significant activity only in the subject‐wise, but not the item‐wise analysis, including lingual and middle occipital gyrus, which would suggest that the activation is due to features of some specific stimuli and that it is not generalizable. The pattern of results was the same in Sample 2 (see Figure 3c,d and Table 1).

#### 3.2.2 Theory of mind

**Figure 4:**
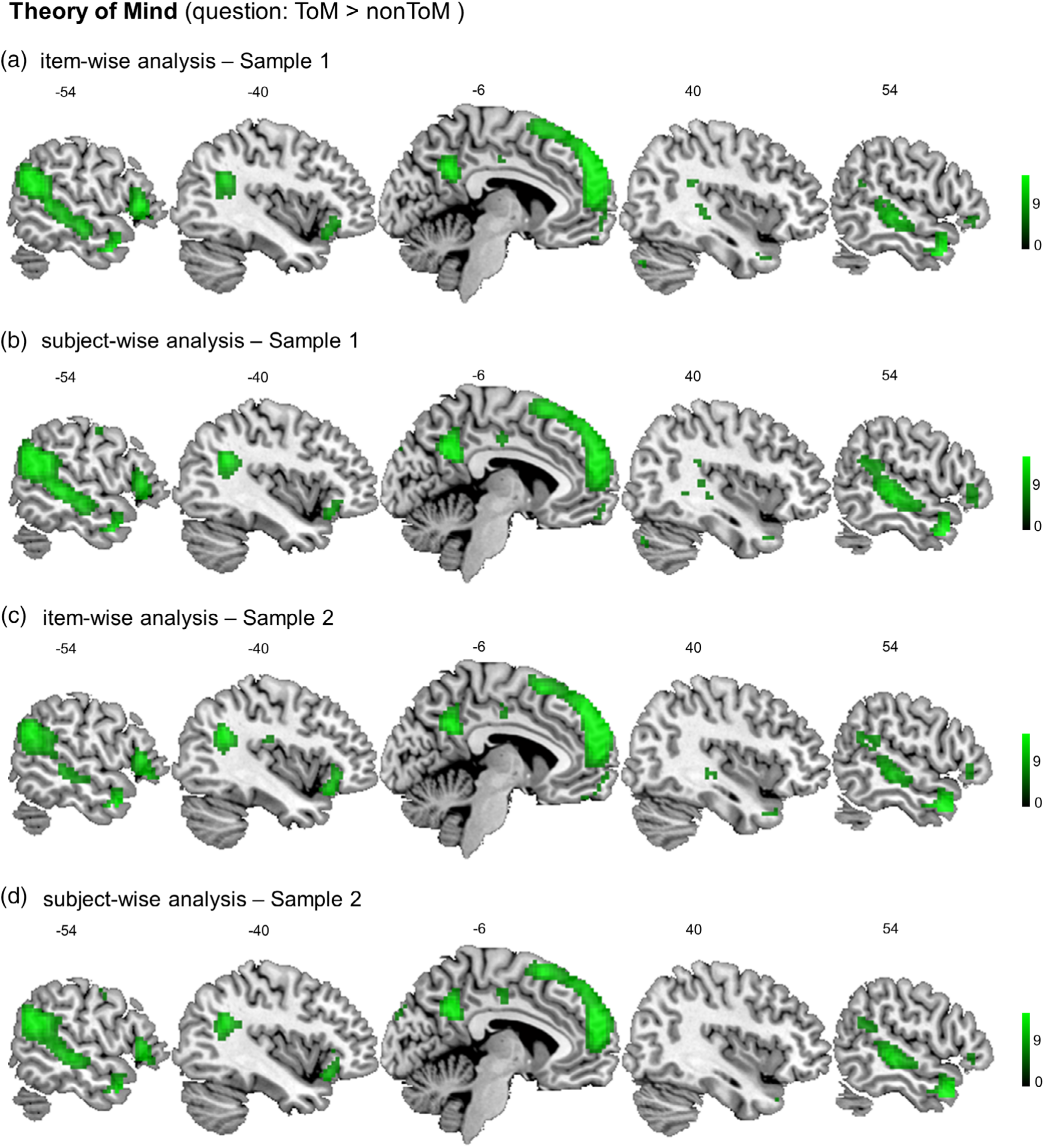
Consistency of the theory of mind related activation patterns (question: ToM > nonToM) across item‐wise (a) and subject‐wise (b) analyses in Sample 1 (*N* = 178) and in Sample 2 (*N* = 130) (c, d, respectively). The results show activity in the theory of mind related network for mental state vs. factual reasoning questions, both across subjects and across items. This network included bilateral ventral temporoparietal junction, superior temporal sulcus, temporal poles, precuneus and anterior medial prefrontal cortex. ToM, Theory of Mind

As for empathy, we first performed whole brain subject‐ and item‐wise random effects analyses on the data set acquired in Sample 1 (see Figure 4a,b and Table 2). All of the brain regions typically involved in ToM were activated for ToM questions compared to factual reasoning questions, both across subjects and across items. These regions include bilateral ventral TPJ, STS, temporal poles, precuneus, and anterior MPFC. Brain regions active for subject‐wise analysis and not item‐wise analysis include parts of the supplementary motor area, postcentral gyrus, and cuneus. The pattern of results was the same in Sample 2 (see Figure 4c,d and Table 2).

#### 3.2.3 Regression analysis

We performed stepwise forward/backward regression analyses on the data set acquired in Sample 2 for the empathy (23 ROIs) and ToM (23 ROIs) contrast. All ROIs were independently defined by the whole‐brain subject‐wise analysis of Sample 1. The results show that for both contrasts condition is almost the only predictor for all regions that were tested (see Table 3). For the ToM contrast, activity in the left cuneus is positively associated with the number of syllables of the questions, the activity in the right cuneus is positively associated with the number of words and the activity in the supplementary motor area could not be explained by either condition or by any other stimulus characteristic. For the empathy contrast, three ROIs (left middle temporal cortex, bilateral middle occipital cortex) could not be associated with any of the predictors. Low‐level factors such as linguistic or general characteristics do not show a major influence regarding the activation across the empathy or ToM network.

**Table 3:** Results of the regression analyses

					Coefficients			
ROI	*H*	*F*	*p*	*R* ^2^	Predictor	*β*	*t*	*p*
*Question ToM* > *non ToM*								
Rectus	*R*	12.034	.001	.093	Condition	.304	3.469	.001
Superior medial frontal	*L*	247.114	<.001	.677	Condition	.823	15.720	<.001
Superior frontal	*L*	203.853	<.001	.633	Condition	.796	14.278	<.001
Superior medial frontal	*R*	147.355	<.001	.555	Condition	.745	12.139	<.001
Inferior frontal gyrus	*R*	35.606	<.001	.232	Condition	.481	5.967	<.001
Inferior frontal gyrus	*L*	120.307	<.001	.505	Condition	.711	10.968	<.001
Inferior frontal gyrus	*L*	147.889	<.001	.556	Condition	.746	12.161	<.001
Temporal pole	*R*	223.881	<.001	.655	Condition	.809	14.963	<.001
Temporal pole	*L*	159.097	<.001	.574	Condition	.758	12.613	<.001
Postcentral	*L*	12.593	.001	.096	Condition	.311	3.549	.001
Middle cingulate		28.239	<.001	.193	Condition	.439	5.314	<.001
Supplementary motor area	*R*				None			
TPJ‐middle temporal	*R*	98.618	<.001	.455	Condition	.675	9.931	<.001
TPJ‐superior temporal	*R*	71.325	<.001	.377	Condition	.614	8.445	<.001
TPJ‐angular gyrus	*R*	27.034	<.001	.186	Condition	.432	5.199	<.001
Posterior cingulate/precuneus	*L*	107.008	<.001	.476	Condition	.690	10.344	<.001
TPJ‐angular gyrus	*L*	148.454	<.001	.557	Condition	.753	12.184	<.001
TPJ‐middle temporal	*L*	103.657	<.001	.468	Condition	.684	10.181	<.001
TPJ‐superior temporal	*L*	59.992	<.001	.337	Condition	.581	7.745	<.001
Cuneus	*L*	15.237	<.001	.137	Syllables	.354	4.294	<.001
				.207	Condition	.264	3.203	.002
Cuneus	*R*	16.476	<.001	.123	Words	.350	4.059	<.001
Cerebellum	*L*	143.531	<.001	.549	Condition	.741	11.980	<.001
Cerebellum	*R*	165.993	<.001	.584	Condition	.765	12.884	<.001
*Video emotional* > *non emotional*								
Inferior frontal gyrus	*L*	58.877	<.001	.333	Condition	.577	7.673	<.001
Middle frontal	*L*	18.694	<.001	.137	Condition	.370	4.324	<.001
Anterior insula	*L*	36.633	<.001	.237	Condition	.487	6.052	<.001
Superior medial frontal	*L*	59.271	<.001	.334	Condition	.578	7.699	<.001
Superior medial frontal	*R*	19.299	<.001	.141	Condition	.375	4.393	<.001
Inferior frontal gyrus	*R*	53.658	<.001	.313	Condition	.559	7.325	<.001
Middle frontal	*R*	13.047	<.001	.100	Condition	.316	3.612	<.001
Anterior insula	*R*	37.890	<.001	.243	Condition	.493	6.156	<.001
Ventral striatum	*R*	17.745	<.001	.131	Condition	.362	4.212	<.001
Ventral striatum	*L*	33.096	<.001	.219	Condition	.468	5.753	<.001
Caudate	*L*	12.161	.001	.093	Condition	.306	3.487	.001
Caudate	*R*	10.419	.002	.081	Condition	.285	3.228	.002
Middle cingulate		8.856	.004	.070	Condition	.264	2.976	.004
Middle temporal cortex	*L*				None			
TPJ‐angular/supramarginal	*R*	17.682	<.001	.130	Condition	.361	4.205	<.001
Middle temporal cortex	*R*	15.180	<.001	.114	Condition	.338	3.896	<.001
TPJ‐angular/supramarginal	*L*	78.740	<.001	.400	Condition	.633	8.874	<.001
Precuneus		12.004	.001	.092	Condition	.304	3.465	.001
Lingual gyrus	*L*	8.807	.004	.069	Condition	.264	2.968	.004
Middle occipital	*R*				None			
Middle occipital	*L*				None			
Cerebellum	*L*	45.261	<.001	.277	Condition	.527	6.728	<.001
Cerebellum	*R*	39.490	<.001	.251	Condition	.501	6.284	<.001

Abbreviations: ROI, regions of interest; ToM, Theory of Mind; TPJ, temporoparietal junction/supramarginal gyrus.

## 4 DISCUSSION

The present study aimed to probe the generalizability and reproducibility of the neural networks related to empathy and ToM. The results demonstrate replicability of subject‐ and item‐wise analyses of both functions with AI, ACC/DMPFC, IFG, dorsal TPJ/SMG for empathy and ventral TPJ, STG/STS, temporal poles and anterior and posterior midline regions for ToM, arguing for generalizability of the brain activation patterns to the respective stimulus classes. Importantly, the observed activity was not predicted by low‐level stimulus characteristics such as the number of words or syllables, corroborating the validity of the activation patterns. Furthermore, we used the item information to construct stimulus sets that include the most effective items to provide the most stable and reproducible results in future studies employing the EmpaToM paradigm. Lastly, demonstrating reproducibility of the findings, all of the above described results were replicated in a second, independent participant sample.

The main result of the present study is the finding of consistent activation patterns for empathy and ToM across subject‐ and item‐wise analyses. This consistency demonstrates that the observed network activity is not due to idiosyncratic characteristics of (some of) the utilized videos and questions, but is generalizable to the entire populations of stimuli. One critical question here is what exactly defines these stimulus populations. Just as the generalizability of subject‐wise analyses is limited by how well the participant sample represents the population (e.g., the age range of 20–55 years in the present study precludes conclusions about empathy and ToM processing in older adults), generalizing the results to empathy inducing or ToM demanding situations needs to be done with care, considering the breadth of situations covered in the applied stimuli. The shown videos were created to resemble brief episodes of a (putatively longer) complex conversation one might have with another person. The ToM questions ask for aspects of the mental state of this person that were not overtly described. While this enables generalizing to the empathic sharing of others' affect as conveyed in language, prosody and facial expression, it precludes generalizing to other forms in which people express their emotions, such as gesture, body posture and movement or the direct observation of emotional situations, for instance, of injury. Moreover, the emotional videos in the EmpaToM paradigm are negatively valenced which also precludes generalizing to positive empathy, that is, sharing, or joining others' positive emotions. The theory of mind questions aim at an understanding of a person's mental states. Stimuli that target the prediction of a person's behavior are not included in this task. In comparison to other tasks in theory of mind, the items cannot generalize to mental state attributions that are based on action observation as in social animations (e.g., Castelli et al., 2000), or to conceptual knowledge about persons as in trait judgments (e.g., Mitchell et al., 2002). Consequently, the stimuli of the EmpaToM task do not elicit all possible forms of empathic responses and theory of mind reasoning. A more comprehensive approach to generate a random sample of items that is representative for theory of mind and empathy might be realized by an ecological momentary assessment (EMA) (Shiffman, Stone, & Hufford, 2008). This approach involves repeated sampling of subject's social interactions in real time over periodic intervals, thereby enabling a high ecological validity. Future studies could therefore arrive at stronger conclusions about the precise nature of the population of items.

However, given the amount of videos and questions (240 in total for each type) and the fact that no situation was repeated, there is considerable breadth within this conversation type situation. Complying with the call for “item‐analyses with a larger and more variable set of stimuli” (Dodell‐Feder et al., 2011), the present results, thus, expand previous reports of consistent activity for reading false‐belief (20 items; (Dodell‐Feder et al., 2011)) and physical or emotional pain stories (24 items each; (Bruneau et al., 2013)).

Another critical question pertains to possible confounds due to the, in general, high error rates and the differences in behavioral performance. The EmpaToM task was explicitly designed to be hard, which makes it unique among other theory of mind tasks in functional neuroscience in adults. In other tasks, for example, false belief or social animations, healthy participants perform typically at 100% or nearly 100% accuracy. The drawback of those measurements is that they are not sensitive to pick up improvements in performance over time, whereas the EmpaToM task can (Böckler et al., 2017; Trautwein et al., 2020). Given that participants were less accurate in the nonToM condition than in the ToM condition, one might think that the differential brain activation identified with the contrast (ToM > nonToM) reflects the effect of general task difficulty. However, we think this is unlikely because of the following reasons: First, prior to the fMRI measurements, participants were sufficiently familiarized with the task and the different conditions. Second, a previous study that validated the EmpaToM task with other measures of empathy and theory of mind did not detect any differences in accuracy (Kanske et al., 2015; Exp. 1). In line with these results, subjects' confidence ratings, indicating their performance evaluation, were equal across all conditions, meaning the participants did not evaluate the nonToM condition as more difficult than the ToM condition. Finally, further results of this study also showed that the theory of mind performance does positively correlate with the activity of the default mode network, whereas areas in the default mode network typically tend to increase in deactivation with increasing task difficulty (e.g., Buckner, 2008).

Activity in a few regions observed in the subject‐wise analyses was not present for the item‐wise analyses. These include the supplementary motor area, postcentral gyrus, and cuneus for ToM and lingual and middle occipital gyrus for empathy. The results of the regression analyses could partly explain this difference by showing that activity in the bilateral cuneus was mainly due to the number of syllables and words of the theory of mind and factual reasoning questions and not the condition difference itself. The lack of activation in the other areas in the item‐wise analyses suggests that their subject‐wise activation is due to specifics of the videos and questions used, implying that they would not be activated by other empathy and ToM stimuli. This is in line with the absence of these regions in empathy and ToM meta‐analyses (Bzdok et al., 2012; Lamm, Batson, & Decety, 2007; Schurz et al., 2014).

FMRI item‐analyses allow an item‐specific estimate for the neural activity in a brain region which might serve as an indicator of the regions function. As the items can be characterized not only regarding their experimental category but also regarding multiple other features (e.g., constituent size, or syntactic complexity), it is possible to determine which features best predict the neural response in each brain region (see e.g., Bruneau et al., 2013; Dodell‐Feder et al., 2011). This allowed us to test whether low‐level stimulus characteristics, which might confound the manipulation of empathy and ToM, have contributed to some of the activations attributed to the experimental condition. The results of the regression analyses yielded the experimental condition as strongest predictor (by far) for all of the observed activation clusters, demonstrating convincingly that none of the low‐level predictors exert major influence on the results. As it is impossible to completely match emotional and neutral videos without erasing the difference in emotionality, this is an important, reassuring finding. Also with regard to ToM, ruling out the possibility that linguistic characteristics account for the ToM effects is important, because of the considerable overlap of ToM related activity with regions involved in language processing, particularly in the temporal cortex and TPJ (Friederici, 2011; Molenberghs, Johnson, Henry, & Mattingley, 2016; Schurz et al., 2014) and the discussion of the intricate relationship of ToM and language processing (de Villiers & Pyers, 2002; Ferstl & von Cramon, 2002). The results of the regression analyses showed that low‐level features do not explain the neural response in the ToM or empathy regions. A different approach could also focus on high‐level features, such as whether the ToM questions include true or false belief, or first or second order reasoning. This approach might, therefore, be of particular importance for future research on social cognition identifying areas with specific functions for ToM and empathy processing.

Given the recent discussions about difficulties in replicating psychological findings (Lindsay, 2015; Open Science Collaboration, 2015), we aimed at testing the stability of our findings in a within‐study replication. Indeed, the results from a second independent sample corroborated the conclusions of the first sample, that is, reproducible neuroimaging results in subject‐ and item‐wise analyses that are independent from low‐level stimulus characteristics. Furthermore, addressing the critique of small sample sizes in many neuroimaging studies (Button et al., 2013), the two samples we assessed were relatively large in comparison to most fMRI investigations (which mostly include <40 participants) (David et al., 2013). Thus, the present study lends a high degree of trustworthiness to the observed neural activation patterns for empathy and ToM. Future studies could of course further strengthen this conclusion, for instance by probing the test–retest‐reliability of the results, which has been shown to be highly variable across brain regions and experimental paradigms (Plichta et al., 2012).

The specific activation patterns observed for empathy and ToM are not only consistent across subject‐ and item‐wise analyses, but also correspond to the typical networks associated with the two functions in large‐scale meta‐analyses (Bzdok et al., 2012; Lamm et al., 2011; Molenberghs, Johnson, et al., 2016; Schurz et al., 2014). An interesting aspect is that the meta‐analyses suggest the existence of core networks for empathy (AI, IFG, ACC) and ToM (TPJ, MPFC), activated for all operationalizations of the respective functions, and extended networks that include additional regions (for empathy: DMPFC, dorsal TPJ/SMG; for ToM: STG/STS, temporal poles, precuneus), when pooling across the different operationalizations. Assuming that most experimental paradigms capture specific component processes of full‐fledged empathy or ToM (Schurz & Perner, 2015), the finding of activation in the extended networks for the EmpaToM suggests that the task comprehensively captures the complexity of these two social capacities (as is the case for other paradigms aiming at ecological validity (Wolf, Dziobek, & Heekeren, 2010)). Furthermore, taking the independence of the neural bases of empathy and ToM into account (Kanske et al., 2015; Kanske et al., 2016) and observing the two networks in both types of analyses here, corroborates the assumption that empathy and ToM are distinct social functions, possibly serving specific purposes in social encounters, for example, establishing the motivation for cooperation and enhancing prosocial behavior (Kanske, Bockler, & Singer, 2017; Tusche, Bockler, Kanske, Trautwein, & Singer, 2016).

The results of the item‐analysis made it possible to select those videos and questions that elicit the most prototypical responses in terms of activation in the neural networks that meta‐analyses have associated with empathy and ToM (Bzdok et al., 2012; Lamm et al., 2011; Schurz et al., 2014) and in behavior. To avoid circularity, we selected the stimuli based on Sample 1 and tested them in the independent Sample 2, showing strong and consistent activation patterns across the two samples. This way, we could form several optimized stimulus sets for future usage in specific settings. In particular, the short versions of the task enable testing special populations with reduced attention spans, for instance, in psychopathology (Preckel, Kanske, Singer, Paulus, & Krach, 2016) or assessing multiple tasks, including the EmpaToM, within one session, for instance, to predict social behavior based on empathic and ToM capabilities (Tusche et al., 2016). The optimized parallel sets could be applied in longitudinal designs, including intervention research.

To conclude, by replicating the empathy and ToM related neural networks across item‐ and subject‐wise analyses and demonstrating their independence from low‐level stimulus characteristics, the present results contribute methodologically to the social neuroscience literature and add to our understanding of these social capacities as distinct functions.

## AUTHOR CONTRIBUTIONS

Conceptualization, all authors; Data curation, F.M.T., A.B.R., P.K.; Formal analysis, M.G.T., P.K., F.M.T.; Funding Acquisition, T.S.; Investigation, F.M.T., A.B.R., P.K.; Methodology, F.M.T., A.B.R., P.K.; Project administration, T.S., F.M.T., A.B.R., P.K.; Resources, T.S.; Supervision, T.S., P.K.; Validation, M.G.T.; Visualization, M.G.T.; Writing—original draft preparation, P.K., M.G.T.; Writing—review and editing, all authors.

## Supporting information


**Appendix S1**: Supporting information

## Data Availability

The data of this study are available from the authors upon reasonable request.

## Endnotes

1 Please note that sample 1 in the present study is based on the same participant sample as described in Kanske et al. (2015). Importantly however, the analyses and results described in the present study are novel and have not been described or shown elsewhere.

2 In contrast to empathy, compassion is defined as feelings of warmth and care, including the motivation to improve the other's wellbeing (Singer & Klimecki, 2014).
